# Chronic stress increases the susceptibility to chronic vulvar pain following acute vulvar inflammation in a preclinical model of vulvodynia

**DOI:** 10.1186/s13041-026-01277-3

**Published:** 2026-02-07

**Authors:** Yaseen Awad-Igbaria, Saher Abu-Ata, Renad Jabarin, Reem Sakas, Leqaa Igbaria, Ayah N. Hamdan, Lior Lowenstein, Jacob Bornstein, Eilam Palzur, Alon Shamir

**Affiliations:** 1 Azrieli Faculty of Medicine in the Galilee, Bar-Ilan University, Safed, Israel; 2 Research Institute of Galilee Medical Center, Nahariya, Israel; 3 Sagol Department of Neurobiology, Faculty of Natural Sciences, University of Haifa, Haifa, Israel; 4 Pediatric Department at Hadassah Ein-Kerem, Jerusalem, Israel; 5 Department of Anesthesiology, Center for Clinical Pharmacology, Washington University Pain Center, Washington University in St. Louis, St. Louis, MO USA; 6 Department of Obstetrics and Gynecology, Galilee Medical Center, Nahariya, Israel; 7 Psychobiology Research Laboratory, Mazor Mental Health Center, Akko, Israel; 8 Ruth and Bruce Rappaport Faculty of Medicine, Technion–Israel Institute of Technology, Haifa, Israel

**Keywords:** Anxiety, Chronic pain, Depression, Mood disorder, Provoked vulvodynia

## Abstract

Psychological distress and chronic stress were suggested to contribute to the pathophysiology of idiopathic pain conditions such as provoked vulvodynia (PV). The comorbidity of PV and mood disorder is quite common. Thus, vulvar pain can trigger anxiety, and mood disruption, whereas elevated anxiety and mood disruption play a critical role in pain maintenance. Yet, whether chronic stress can facilitate the development of chronic vulvar pain remains unclear. Here, we aimed to assess the effects of chronic stress on anxiety, depression-like behaviors, and the development of chronic vulvar pain after vulvar inflammation, which combines acute inflammation with chronic unpredictable stress (CUS) in female rats. Current result indicates that CUS leads to a reduction in vulvar mechanical thresholds and an increase in anxiety-like behavior, including reduced entries and time spent in the open arms of the EPM, reduced time in the center, increased distance moved in the OF, and reduced sucrose intake compared to the non-CUS group. Blood corticosterone levels and gene expression related to neuronal activation (cFOS) and GABA-synthesis (GAD67) were significantly increased in the amygdala and PAG in the CUS group compared to the non-CUS group. Following vulvar injection (saline/zymosan), there was a significant reduction in vulvar mechanical threshold in all groups: non-CUS/Saline, non-CUS/Zymosan, CUS/Saline, and CUS/Zymosan. However, mechanical thresholds returned to baseline in all groups except the CUS/Zymosan group, which exhibited prolonged vulvar hypersensitivity with no sign of recovery. Long-term behavioral assessments revealed reduced open-arm entries, altered locomotion, and decreased sucrose intake of the CUS groups compared to non-CUS groups. In conclusion, chronic stress enhances vulnerability to chronic vulvar pain following acute inflammation, alongside persistent anxiety and depression-like behaviors. These findings support a biopsychosocial model of PV, emphasizing the interplay between stress and inflammation in vulvar pain chronification.

## Introduction

Chronic vulvar pain can result from nerve injury, trauma, and inflammation that contribute to peripheral nociceptor sensitivity [1–3]. However, some vulvar pain conditions, such as provoked vulvodynia (PV), are considered idiopathic pain disorders (IPD), which have no clear identifiable cause [4]. PV affects 7%-15% of women [5], and it’s characterized by hypersensitivity, allodynia, and severe pain of the vulvar vestibule upon an attempt of vaginal penetration (e.g., intercourse, tampon use) [6–9]. The pathogenesis of IPD, especially PV, is poorly understood, and the etiology is multifactorial, involving genetic, hormonal, and immunological factors [10–15].

Psychological distress was also suggested as a factor in the development of PV [16–19]. Thus, previous studies have shown that traumatic events, abuse, PTSD can contribute to vulvodynia and vulvar pain [16–19]. For-instance, women with depression and PTSD had a 50% and 20% higher prevalence of vulvodynia, respectively [20]. In addition, childhood maltreatment and child sexual abuse are considered as a risk factor in the development of vulvodynia [16]. Yet, how psychological distress increases the risk for vulvar pain development is still unclear, and more research needs to be adequately applied to address this issue [21].

Interestingly, a growing body of evidence suggests that a central mechanism related to dysfunction of endogenous analgesia underlies the neuronal mechanism of idiopathic pain conditions including PV [4, 22]. Endogenous pain modulation/control involves descending pathways that directly or indirectly project to the dorsal horn of the spinal cord (DHSC) [23–25]. Recent studies suggest that stress disrupts the normal activity of several brain networks involved in descending pain modulation and mood regulation, including monoaminergic neurons in the brainstem nuclei (locus coeruleus, rostral ventromedial medulla, dorsal raphe nucleus), the periaqueductal grey (PAG), and the amygdala [26–29]. The balanced activity of the PAG, which is the primary control center for descending pain modulation and relief, is essential for mood and pain regulation [28, 30–32]. Hence, dysfunction of the PAG activity has been reported in mood disorders and chronic pain and conditions [27, 33]. Interestingly, previous findings suggest that an imbalance in the PAG-excitatory and inhibitory signaling as a result of chronic stress or laboratory-pharmacological intervention elicits depression-like behavior and mechanical allodynia [27, 33, 34].

Furthermore, the amygdala is believed to be a critical component of the pain and mood circuits [35]. Previous studies have shown that dysfunction of the amygdala activity contributes to imbalance in the descending pain pathway [30, 36]. In addition, several studies have shown abnormal amygdala hyperactivity in chronic pain disorders in humans and laboratory animals [37, 38]. Due to amygdala dysfunction in chronic pain, anxiety-like behavior and depression are very expected. Indeed, up to 50% of patients with chronic pain exhibit symptoms of anxiety and depression [39]. Notably, the presence of depression and anxiety has been found to lead to an extended duration and heightened intensity of pain, thereby fostering a self-perpetuating cycle of pain and mood disorder symptoms [40, 41]. These findings support the notion that psychological distress and chronic stress, which probably leads to amygdala dysfunction, also contribute to the pathophysiology of chronic vulvar pain when there is no clearly identifiable cause [4, 18]. However, it is still unclear whether alterations in pain and mood circuits (e.g., PAG, amygdala), due to chronic stress, facilitate the transition from acute to chronic vulvar pain following acute inflammation. Accordingly, we aimed to examine the short and long-term effects of chronic stress on anxiety, depression-like behaviours, and chronic vulvar pain development following vulvar inflammation in a well-established vulvar model in rats [42–45].

## Result

### CUS induces peripheral hypersensitivity in female rats

**Fig. 1:**
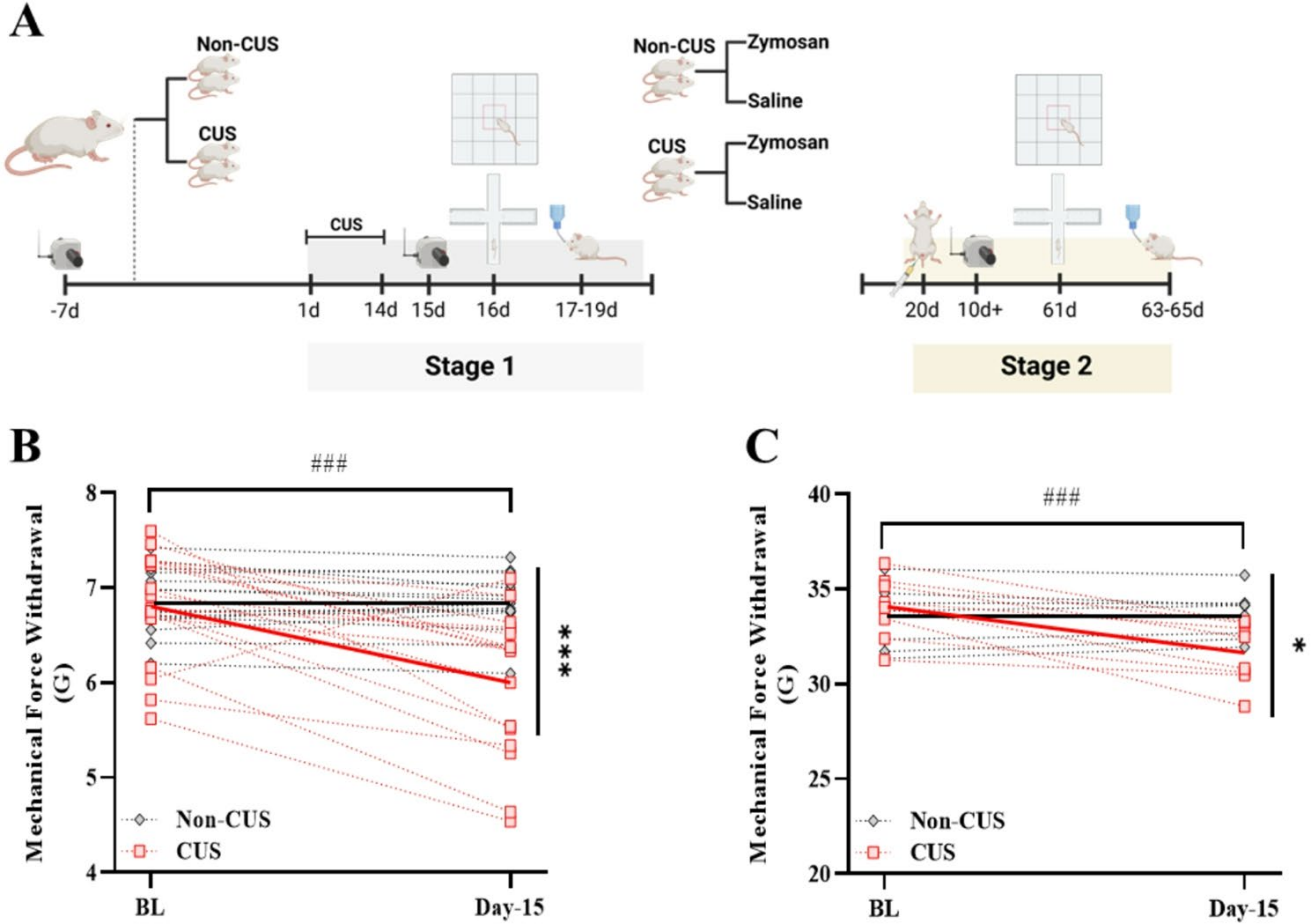
Chronic unpredictable stress leads to peripheral hypersensitivity. **A** The experimental timeline. In stage one, after measuring vulvar and hind paw mechanical sensitivity, rats were randomly assigned to one the following groups: non-CUS and CUS group. The CUS paradigm was performed between day 1 to 14. Behavioral test: VF, EPM, OF and SPT, were performed between day 15 to 19. In stage two (day 20), rats were randomly assigned to one the following groups: non-CUS/Saline, non-CUS/Zymosan, CUS/Saline, CUS/Zymosan. Vulvar rat was challenged with zymosan or saline on day 20. Vulvar mechanical sensitivity was measured every 5–10 days after the zymosan/saline challenge. Behavioral test: EPM, OF, SPT and SIT were performed between day 61 to 68, i.e., 41–48 days after the vulvar challenge. **B** Vulvar mechanical force withdrawal (G) of the non-CUS and the CUS group. **C** Hind paw mechanical force withdrawal (G) of the non-CUS and the CUS group. (*n* = 8–16 per group). Mixed Model ANOVA; Followed with Student t-test. Mean ± SEM. ^###^*P <* 0.001 Compared to Baseline; ^*^P < 0.05, ^**^P < 0.05, ^***^P < 0.001 Difference between groups

Peripheral hypersensitivity of the vulvar and hind paw was assessed before and after chronic unpredictable stress (CUS) induction (Fig. 1A). The analysis revealed that CUS leads to peripheral hypersensitivity development. Thus, a significant reduction in vulvar mechanical threshold [*Time*: F_(1,29)_ = 19.23, *P* = 0.00013, η^2^ = 0.39; *Group*: F_(1,29)_ = 6.64, *P* = 0.015, η^2^ = 0.186; *Time X Group*: F_(1,29)_ = 16.84, *P* = 0.0003, η^2^ = 0.367, Fig. 1B], and hind paw mechanical threshold was noted in the CUS group compared to the baseline and the non-CUS group [*Time*: F_(1,14)_ = 26.97, *P* = 0.00013, η^2^ = 0.65; *Group*: F_(1,14)_ = 1.01, *P* = 0.331, η^2^ = 0.068; *Time X Group*: F_(1,14)_ = 32.25, *P* = 0.00005, η^2^ = 0.69, Fig. 1C]. Suggesting that CUS can lead to an increased peripheral mechanical hypersensitivity.

### Increased level of anxiety and depression-like behavior following CUS

**Fig. 2:**
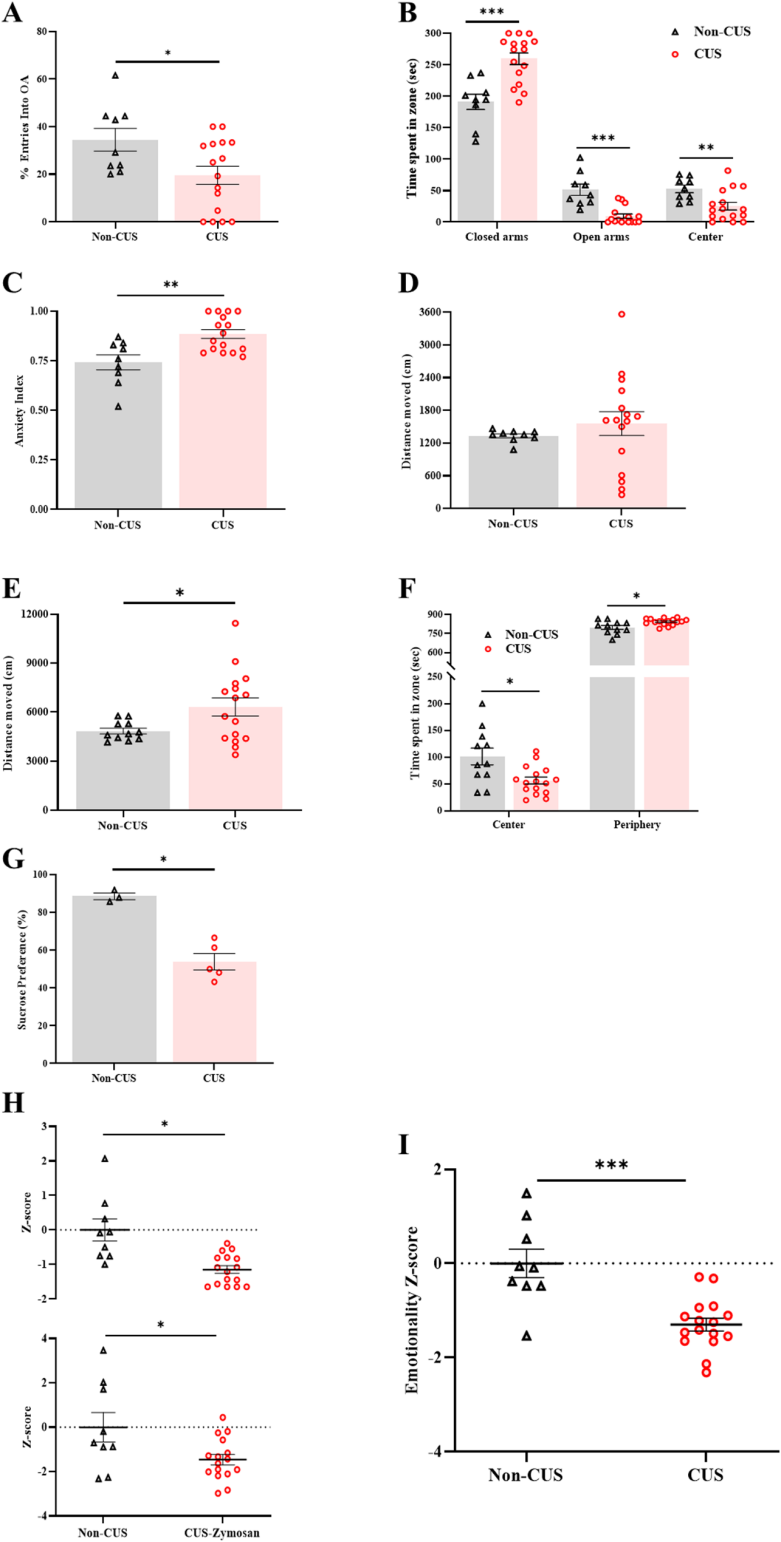
Anxiety and depression-like behaviors following two weeks of CUS. **A** The percentage of open-arms entries. (**B**) Time spent in the closed arms of the EPM (Second). **D** Time spent in the open arms of the EPM (Second). **C** The EPM-Anxiety index (0 = low anxiety level; 1 = high anxiety levels). **D** Distance moved in the EPM (cm). **E** Distance moved in the OF (cm). **F** Time spent in the center and the periphery in the OF (Second). **G** Sucrose preference index. **H** Normalization of data using z-score method was performed for EPM (Upper panel) and OF parameters (Lower panel). **I** The integrated total emotionality Z-scores. Behavioral assessment (*n* = 8–16 per group). Student t-test. Mean ± SEM. ^*^P < 0.05, ^**^P < 0.05, ^***^P < 0.001 Difference between groups

To confirm that our CUS paradigm induces anxiety and depression-like behavior, the behavioral tests were conducted after two weeks of CUS (Fig. 1A). The analysis revealed that the CUS group expressed higher levels of anxiety. Thus, the CUS group avoids the entrance to the open arms in the EPM (t_(23)_ = 2.41, *P* = 0.024, Fig. 2A), and spend less time in the open arms, and more time in the closed arms compared to the non-CUS group [Open arms: t_(23)_ = 5.25, *P* = 0.00002; Closed arms: t_(23)_ = -4.45, *P* = 0.00182; Center: t_(23)_ = 2.92, *P* = 0.00759, Fig. 2B]. Strikingly, the anxiety index analysis of the EPM revealed a significant increase in the anxiety index of the CUS compared to the non-CUS group (t_(23)_ =-3.49, *P* = 0.0020, Fig. 2C). In the OF, higher distance moved was noted in the CUS group to the non-CUS group (t_(25)_ =-2.39, *P* = 0.0204, Fig. 2E). In addition, the CUS group tended on average to spend less time in the center of the area and more time in periphery (Center: t_(25)_ = 2.97, *P* = 0.00634, periphery: t_(25)_ = 2.97, *P* = 0.00640, Fig. 2F).

Furthermore, depression-like behavior and anhedonia symptoms were noted following the CUS. Thus, compared to the non-CUS group the CUS group shows a reduction in the percentage of sucrose intakes (t_(6)_ = 5.814, *P* = 0.00056, Fig. 2G).

Additionally, we performed an emotionality Z-score analysis to investigate the potential of combining results across different behavioral tests for anxiety-like behaviors. The analysis revealed a significant reduction in the emotionality of the CUS group compared to the non-CUS group (Z-score-OF: t_(23)_ = 2.49, *P* = 0.02037; Z-score-EPM: t_(23)_ = 4.11, *P* = 0.00041; Z-score- emotionality: t_(23)_ = 4.53, *P* = 0.00015, Fig. 2H, I).

### Corticosterone level and gene-expression changes following CUS

**Fig. 3:**
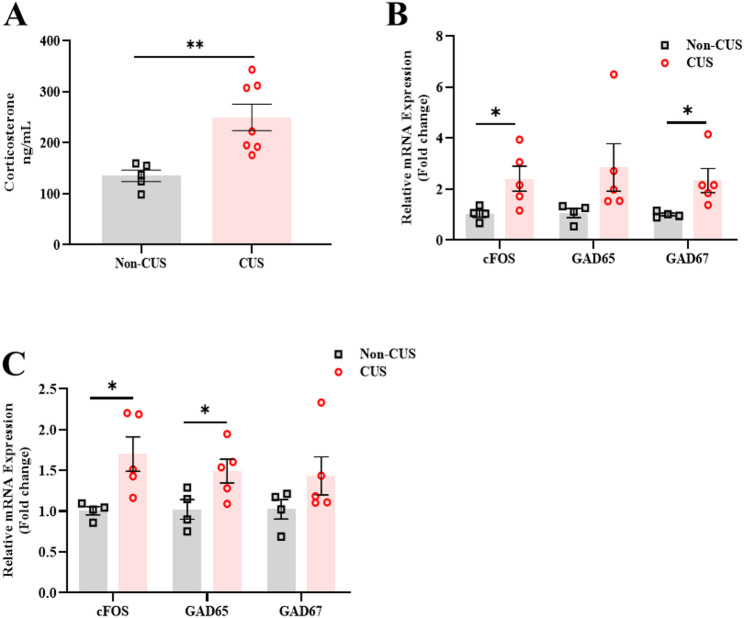
CUS enhances the level of corticosterone and gene-expression in brain regions involved in pain and mood. **A** Corticosterone level in the non-CUS, and CUS (after 1 day of the last CUS challenge). **B** Gene expression in the Amg and, **C** the PAG. (*n* = 4–7 per group). Mean ± SEM. Student *t*-test, ^*^P < 0.05, ^***^P < 0.001

Following two weeks of CUS (Fig. 1A), we assessed the stress marker level corticosterone in the blood. The analysis revealed a significant increase in the corticosterone level in the CUS group compared to the non-CUS group (t_(10)_ = 3.51, *P* = 0.005557, Fig. 3A). In addition, we examined the gene-expression related to neuronal activation and the Gaba synthesis in the amygdala (Amg), and periaqueductal gray (PAG), that are involved in stress, mood and pain regulation. We found that CUS leads to a significant increase in the transcription of the neuronal activation marker c-Fos as well as Gaba synthesis (GAD67) in the Amg (cFOS, t_(7)_ =-2.402, *P* = 0.04707; GAD65, t_(7)_ =-1.670, *P* = 0.13914; GAD67, t_(7)_ =-2.463, *P* = 0.04276, Fig. 3B), and PAG following CUS (cFOS, t_(7)_ =-2.857, *P* = 0.02444; GAD65, t_(7)_ =-2.384, *P* = 0.04861; GAD67, t_(7)_ =-1.443, *P* = 0.19357, Fig. 3C).

### CUS increases the susceptibility to chronic vulvar pain development following acute vulvar inflammation

**Fig. 4:**
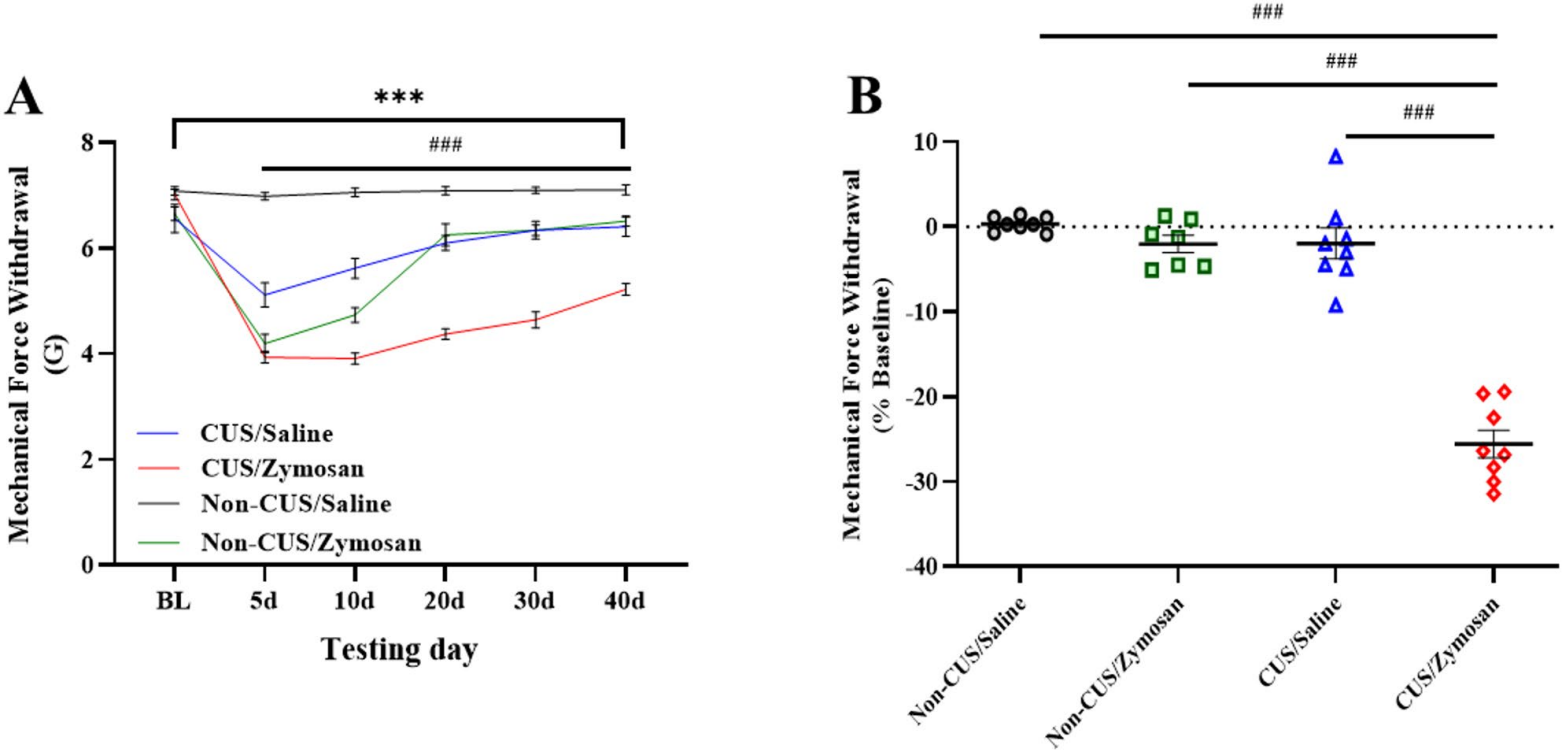
CUS increases susceptibility to chronic vulvar pain development. **A** The vulvar mechanical force withdrawal (G) of the non-CUS/Saline, non-CUS/Zymosan, CUS/Saline, CUS/Zymosan group following the injection of saline or zymosan in the vulvar on day 20. **B** The vulvar mechanical force withdrawal test of day 40 was normalized to the baseline. Behavioral assessment (*n* = 7–8 per group). Mixed Model ANOVA; One-way ANOVA Followed with Tukey test. Mean ± SEM. ^###^*P <* 0.001 Compared to Baseline; ^***^P < 0.001 Difference between groups

Following the CUS procedure, rats were randomly assigned to one of the vulvar injection groups: non-CUS/Saline, non-CUS/Zymosan, CUS/Saline, and CUS/Zymosan. The saline or zymosan was injected in the vulvar on day 20 (i.e., 5 days after the last CUS stimulus, Fig. 1A). Five days after the saline or zymosan injection all groups show a significant reduction in the vulvar mechanical threshold [*Time*: F_(5,135)_ = 205.60, *P* < 0.00001, η^2^ = 0.884; N*on-CUS/CUS*: F_(1,27)_ = 68.33, *P* < 0.00001, η^2^ = 0.717; *Saline/Zymosan*: F_(1,27)_ = 106.96, *P* < 0.00001, η^2^ = 0.798, Fig. 4A].

However, different recovery patterns of the vulvar mechanical threshold was observed following saline or zymosan injection in the vulvar, thus a significant interaction was observed between vulvar mechanical threshold, vulvar injection (saline or zymosan), and the stress experience (Non-CUS or CUS) [*Time X Vulvar injection*: F_(5,135)_ = 69.71, *P* < 0.00001, η^2^ = 0.721; *Time X Stress experience*: F_(5,135)_ = 27.21, *P* < 0.00001, η^2^ = 0.502; *Time X Vulvar injection X Stress experience*: F_(5,135)_ = 34.57, *P* < 0.00001, η^2^ = 0.561, Fig. 4A]. Accordingly, following a single zymosan-induced vulvar inflammation challenge, among rats that were previously exposed to CUS (CUS/zymosan, Fig. 4A), a reduced vulvar mechanical sensitivity threshold was maintained for 40 days (Fig. 4A), in contrast to rats without prior chronic stress exposure (non-CUS/zymosan), whose vulvar mechanical sensitivity threshold recovered to baseline levels within 20 days of the vulvar inflammation challenge (Fig. 4A). Notably, following saline injection in the vulvar, among rats that were previously exposed to CUS (CUS/Saline, Fig. 4A) a reduced vulvar mechanical sensitivity threshold was maintained for 20 days (Fig. 4A), in contrast to rats without prior chronic stress exposure (Non-CUS/Saline), whose vulvar mechanical sensitivity threshold recovered to baseline levels within 5 days of the vulvar inflammation challenge (Fig. 4A). Overall, the vulvar mechanical sensitivity threshold recovered to baseline for the non-CUS/Saline, non-CUS/zymosan, CUS/ Saline group, but not in the CUS/Zymosan group [F_(3, 27)_ = 84.59, *P* = 0.00001, Fig. 4B].

### CUS combined with chronic vulvar pain leads to long-term of anxiety and depression-like behaviors

**Fig. 5:**
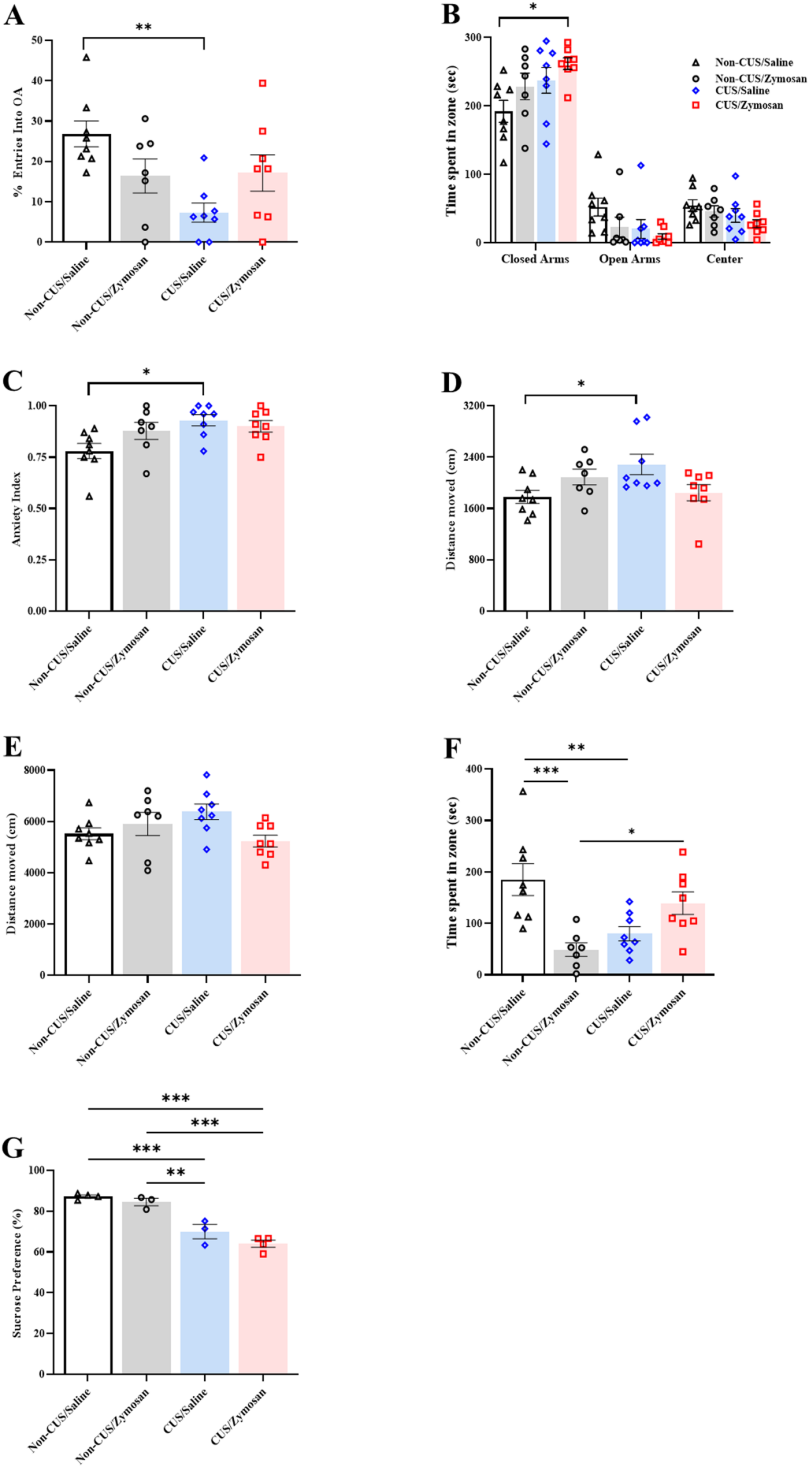
Anxiety and depression-like behaviors following acute vulvar inflammation combined with/without CUS. **A** The percentage of open-arms entries. **B** Time spent in the closed arms of the EPM (Second). **D** Time spent in the open arms of the EPM (Second). **C** The EPM-Anxiety index (0 = low anxiety level; 1 = high anxiety levels). **D** Distance moved in the EPM (cm). **E** Distance moved in the OF (cm). **F** Time spent in the center and the periphery in the OF (Second). **G** Sucrose preference index. Behavioral assessment (*n* = 7–8 per group). Two-way ANOVA, Followed with Student t-test. Mean ± SEM. ^*^P < 0.05, ^**^P < 0.05, ^***^P < 0.001 Difference between groups

Between day 61 to 68 (i.e., after 41–48 days of the saline/zymosan injection in the vulvar (Fig. 1A) we performed the EPM, OF, and SPT (Fig. 1A). We found a significant main effect of the stress experience and interaction between stress experience and the vulvar injection on the open arms entries ratio [*Stress experience*: F_(1, 27)_ = 6.63, *P* = 0.016, η^2^ = 0.197; *Vulvar injection*: F_(1, 27)_ = 0.007, *P* = 0.93, η^2^ = 0.0001; *Interaction*: F_(1, 27)_ = 7.65, *P* = 0.01009, η^2^ = 0.221, Fig. 5A]. Thus, the CUS groups avoid entries to the open arms compared to the non-CUS groups (Fig. 5A). The interaction was derived by a reduction in the open arms entries ratio of the CUS/Saline group compared to Non-CUS/Saline group (*P* = 0.003, Fig. 5A). In addition, a significant main effect of the stress experience on the time spends in the closed [*Stress experience*: F_(1, 27)_ = 5.97, *P* = 0.021, η^2^ = 0.181; *Vulvar injection*: F_(1, 27)_ = 3.57, *P* = 0.07, η^2^ = 0.117; *Interaction*: F_(1, 27)_ = 0.141, *P* = 0.711, η^2^ = 0.005, Fig. 5B]. Thus, the CUS/Zymosan group spend more time in the closed arms compared to the non-CUS/Saline groups (*P* = 0.021, Fig. 5B).

Moreover, the anxiety index analysis of the EPM revealed a significant main effect of the stress experience on the anxiety index [*Stress experience*: F_(1, 27)_ = 6.42, *P* = 0.017, η^2^ = 0.192; *Vulvar injection*: F_(1, 27)_ = 1.04, *P* = 0.316, η^2^ = 0.037; *Interaction*: F_(1, 27)_ = 3.71, *P* = 0.065, η^2^ = 0.121, Fig. 5C]. Thus, the non-CUS/Saline group expressed lower levels of anxiety compared to the CUS/Saline groups (*P* = 0.017, Fig. 5C).

Regarding the distance moved in the EPM and the OF test, the analysis revealed a significant interaction of the stress experience and the vulvar interaction [EPM distance moved: *Stress experience*: F_(1, 27)_ = 1.0, *P* = 0.326, η^2^ = 0.036; *Vulvar injection*: F_(1, 27)_ = 0.24, *P* = 0.626, η^2^ = 0.009; *Interaction*: F_(1, 27)_ = 8.21, *P* = 0.008, η^2^ = 0.233; OF distance moved: *Stress experience*: F_(1, 27)_ = 0.92, *P* = 0.764, η^2^ = 0.003; *Vulvar injection*: F_(1, 27)_ = 1.51, *P* = 0.229, η^2^ = 0.053; *Interaction*: F_(1, 27)_ = 6.15, *P* = 0.020, η^2^ = 0.186, Fig. 5D, E]. The interaction was derived from an increase in distance travel of the CUS/Saline group in the EPM compared to the non-CUS/Saline (*P* = 0.045, Fig. 5D). Notable, Tukey analysis shows no significant difference in the distance travel the OF.

In the OF, there was a significant interaction of stress experience and vulvar injection on time spend in the center of the arena [*Stress experience*: F_(1, 27)_ = 0.12, *P* = 0.732, η^2^ = 0.004; *Vulvar injection*: F_(1, 27)_ = 3.10, *P* = 0.089, η^2^ = 0.103; *Interaction*: F_(1, 27)_ = 20.18, *P* = 0.00011, η^2^ = 0.428, Fig. 5F]. Thus, the non-CUS/Saline and the CUS/Zymosan spent more time in the center compared to the CUS/Saline and the Non-CUS/Zymosan (Fig. 5F).

Furthermore, depression-like behavior and anhedonia symptoms were noted in the CUS groups. Thus, there was a significant effect of the stress experience on sucrose intakes [*Stress experience*: F_(1, 10)_ = 89.80, *P* = 0.000003, η^2^ = 0.900; *Vulvar injection*: F_(1, 10)_ = 4.838, *P* = 0.052, η^2^ = 0.326; *Interaction*: F_(1, 10)_ = 0.641, *P* = 0.442, η^2^ = 0.060, Fig. 5G]. Thus, the CUS/Saline, and CUS/Zymosan groups have shown a reduction in sucrose intakes compared to the non-CUS/Saline, and non-CUS/Zymosan groups (*P* = 0.00002, Fig. 5G).

## Discussion

The current results indicate that CUS in animals induces peripheral hypersensitivity and increases anxiety- and depression-like behaviors, accompanied by gene expression changes in brain regions implicated in pain and mood regulation, as well as elevated corticosterone levels. More critically, CUS was found to increase the susceptibility to chronic vulvar pain, and mood disturbances following an acute vulvar inflammation challenge. To the best of our knowledge, this is the first study to highlight the synergistic role of psychological stress and acute inflammation in driving vulvar pain development. Furthermore, the current model closely mirrors the clinical condition and confirms the previous findings that women with a history of psychological stress are at increased risk for vulvodynia development after experiencing an acute vulvar inflammatory challenge-one that would typically induce only transient, not chronic pain.

Clinical and preclinical evidence indicates that the severity of vulvar pain is associated with mood disorders, including elevated stress levels, anxiety, and depression-symptoms, suggesting that chronic vulvar pain may contribute to the emergence of affective disturbances [43, 44, 46, 47]. However, whether stress itself plays a causal role in the development of chronic vulvar pain remains unclear. In the present study, we provide compelling evidence that chronic stress induces a state of latent sensitization and priming effects, wherein animals display an exacerbated and prolonged response to acute vulvar inflammation.

The development of chronic vulvar pain is not solely attributable to inflammatory mechanisms [2, 48]. Repeated vulvar inflammation has been consistently shown to cause persistent vulvar pain, unlike acute inflammation, which usually leads to temporary allodynia [43, 49]. Yet, current findings suggest a new mechanism by which chronic stress sensitizes the nociceptive system, facilitating the transition from acute to chronic vulvar pain following an initial inflammatory event. These results support a multifactorial pathophysiological model [50], suggesting that the interplay between peripheral inflammatory processes and central stress-mediated sensitization contributes to both the initiation and maintenance of chronic vulvar pain (provoked vulvodynia).

The prevailing hypothesis suggests that stress leads to latent sensitization via disrupting the normal activity of mood and pain regulation system, including the monoaminergic neurons in the brainstem nuclei (LC, RVM), the PAG, and the amygdala [26–29]. This might explain the bidirectional association between mood disorder and pain symptoms among patients with idiopathic pain disorders [51–53]. Consequently, it is unsurprising that the serotonergic and noradrenergic systems became targets for chronic pain treatment [54–56]. Specifically, the serotonin and norepinephrine reuptake inhibitor (SNRIs) medications [56]. Notably, we have previously substantiated the analgesic and anxiolytic effects of Venlafaxine, an SNRI treatment, in preclinical model of chronic vulvar pain [44].

The amygdala is believed to be a critical component of the pain and mood circuits [57, 58]. Several studies have shown abnormal amygdala hyperactivity in chronic pain disorder in humans and laboratory animals [37, 38]. Due to amygdala dysfunction in chronic pain, anxiety-like behavior and depression are very expected. Indeed, up to 50% of patients with chronic pain exhibit symptoms of anxiety and depression [59, 60]. Notably, the presence of depression and anxiety have been found to lead to an extended duration and heightened intensity of pain, thereby fostering a self-perpetuating cycle of pain and mood disorder symptoms [41, 61]. These findings support the notion that psychological distress and chronic stress, which probably leads to amygdala dysfunction, also contribute to the pathophysiology of idiopathic pain [4, 18] when there is no clearly identifiable cause. Remarkably, previous studies have shown that dysfunction of the amygdala activity contributes to imbalance in the descending pain pathway [30, 36]. Thus, the amygdala controls a significant population of the projections in the PAG directly via the central amygdala [62–64]. Interestingly, previous evidence indicates that an increase in central amygdala output generates pain responses under normal conditions without any tissue pathology in laboratory animals and [35, 65, 66], more importantly, in an animal model of chronic stress [67]. Our result may support these observations; thus we observed an increase in neuronal activation marker (cFOS) and GABA-synthesis in the amygdala following CUS.

The balanced activity of the PAG, which is the primary control center for descending pain modulation and relief, is essential for mood and pain regulation [28, 30–32]. Hence, dysfunction of the PAG activity has been reported in mood disorders and chronic pain and conditions [27, 33]. In addition, our result indicates that CUS leads to changes in activation and GABA-synthesis in the PAG, suggesting an imbalance in the PAG signaling. Interestingly, previous findings suggest that an imbalance in the PAG-excitatory and inhibitory signaling as a result of chronic stress or laboratory-pharmacological intervention elicits depression-like behavior and mechanical allodynia [27, 33, 34]. Probably, the excessive activation of the PAG-GABAergic neurons, which project to the RVM/LC, may impair the descending pain pathway (i.e., reduction in the 5HT and NE release in the spinal cord), leading to an amplification of pain signals in the ascending pathway [27, 36, 68, 69]. Nevertheless, whether chronic stress induces dysfunction in descending pain modulatory pathways that facilitates the development of chronic vulvar pain remains incompletely understood. In the current study, we provide behavioral evidence supporting this assumption. However, comprehensive investigation of the specific neuronal circuits and pathways involved in the priming process is essential to elucidate the underlying mechanisms. We explicitly acknowledge this as a limitation of the present study and emphasize the need for future studies to address this question.

Considering that mood disorders are frequently reported in individuals with chronic vulvar pain [47, 70], the insights derived from the present study provide valuable contributions to understanding the etiology of PV and the role of stress in facilitating its development. Thus. the current findings may have several important clinical implications, including the importance of early identification and management of psychological stress in women at risk for PV [19]. This approach may help reduce their likelihood of developing PV after acute vulvar inflammation caused by candida or infection. Moreover, multidisciplinary approaches that address both inflammatory and psychosocial dimensions, including stress management, cognitive-behavioral therapy, and pharmacological interventions, may improve clinical outcomes in women with or at risk for PV [19]. Notably, the current findings also support the inclusion of stress-related biomarkers (e.g., cortisol level) and psychological assessments in future clinical trials as potential predictors of treatment response and PV vulnerability. Hence, we found that chronic stress induces a robust increase in stress markers, which likely facilitates the development of chronic vulvar pain. Overall, the present study emphasizes the biopsychosocial framework for understanding PV and underscores the importance of integrated clinical strategies that target both peripheral and central mechanisms contributing to chronic vulvar pain.

The strengths of this study include the comprehensive assessment of both pain sensitivity and psychological behaviors across multiple time points, with and without prior chronic stress exposure. Additionally, the vulvar pain model employed in this study effectively induced long-lasting mechanical hypersensitivity following an acute inflammatory challenge. Notably, the persistence of behavioral changes may highlight the translational relevance and utility of the current model, which integrates the combined effects of psychological stress and acute inflammation in the development and maintenance of chronic vulvar pain.

Several limitations of our study should be acknowledged. First, the size of each group was modest, which may influence the interpretation of the findings. Therefore, replications using larger samples are critical to establishing the validity of current findings and inferring their practical implications. Here, we focused on the gene-expression changes in the amygdala, PAG following stress. However, these brain regions are most likely only a part of the pain and mood regulation system. Thus, other brain areas, such as the Insula and ACC, may be even more crucial in this process [71–73]. In the current study, we provide behavioral evidence indicating that chronic stress facilitates the development of chronic vulvar pain. However, the specific neuronal mechanisms underlying this effect remain unclear. This represents a limitation of the present study, and we emphasize the need for future investigations to elucidate the underlying neural pathways and mechanisms. Despite these limitations, our findings suggest that chronic stress enhances vulnerability to chronic vulvar pain following acute inflammation, alongside persistent anxiety and depression-like behaviors and gene-expression changes in brain regions linked to pain and mood regulation. These findings support a biopsychosocial model of PV, emphasizing the interplay between stress and inflammation in pain chronification.

## Materials and methods

### Animals

Female Sprague-Dawley rats (240–280 g; 12 weeks of age) were used in this study. The animals were maintained on a 12 h/12 h light/dark cycle (lights on between 7:00 p.m. and 7:00 a.m.) and housed in an air-conditioned room (temperature 21 ± 1 ◦C, relative humidity 60 ± 10%) with ad libitum access to water and food. All animal procedures were approved by Bar-Ilan University Animal Care Committee.

### Chronic unpredictable stress model (CUS)

Chronic unpredictable stress model was produced as described before [74]. Animals were subjected to repeated unpredictable stressors in a random order for two consecutive weeks (See Table 1).

**Table 1:** Daily schedule of stressors used in the chronic unpredictable stress (CUMS) paradigm

Day 1	Day 2	Day 3	Day 4	Day 5	Day 6	Day 7	Day 8	Day 9	Day 10	Day 11	Day 12	Day 13	Day 14
Cold Environment – 30 min	45° tilted cage – 8 h	Predator odor – 2 h	Social isolation – 4 h	All rats in one cage (eight) – 4 h	No bedding – 10 h	Social isolation – 3 h	Wet bedding – 3 h	Food deprivation – 14 h	Dirty bedding – 8 h	Wet bedding – 4 h	Water deprivation – 10 h	Predator odor – 3 h	No bedding – 2 h
Water deprivation – 8 h	Restraint – 2 h	Wet bedding – 4 h	Cold environment – 20 min	45° tilted cage – 8 h	Restraint – 2 h	Water deprivation – 5 h	All rats in one cage (eight) – 3 h	Predator odor – 1.5 h	Social isolation – 5 h	Food deprivation – 10 h	No bedding – 6 h	45° tilted cage – 8 h	Restraint – 1 h
Dark – 16 h	No bedding – 8 h	Food deprivation – 5 h	Small cage – 4 h	Water deprivation – 8 h	Dirty bedding – 4 h	Dark – 20 h	45° tilted cage – 4 h	Restraint – 2 h	Cold environment – 45 min	Light – 18 h	Cold environment – 25 min	Social isolation – 4 h	Dirty bedding – 6 h
	Stroboscopic lighting – 12 h			Predator odor – 1 h			Stroboscopic lighting – 12 h						

### A rat model of vulvar pain

The vulvar pain model was produced using an acute inflammation challenge by zymosan [42]. Single injection of zymosan (300ul, 10 mg/ml) or saline (as control, 300ul) into the vulva was carried out under isoflurane anesthesia. Based on our previous study [43], an acute vulvar inflammation challenge induces transient vulvar pain that persists for approximately two weeks following zymosan injection.

### Behavioral tests and pain assessment

The behavioral tests were performed under red light illumination, during the dark phase of the cycle. The tests were recorded and analyzed using a computer-controlled tracking system (EthoVision XTV.15).

#### Mechanical sensitivity testing

Mechanical sensitivity was assessed using an electronic Von Frey (VF) device (cat. No.38450 Ugo Basile, Italy). After thirty minutes of acclimatization in the testing chambers, five values were collected by an observer blind to the experimental condition for each rat.

#### Elevated plus-maze

The elevated plus maze (EPM) was produced as described before [75]. The session lasted for 5 min, time spent and frequency of visits to the different zones were recorded and measured automatically.

#### Open field test

The open field (OF) test was produced as described before [76, 77]. The session lasted 15 min, and measurements of locomotor activity and time spent in the center of the arena were quantified.

#### Sucrose preference test

The sucrose preference test was produced as described before [78]. Briefly, animals were habituated to the presence of two drinking bottles of 1% sucrose solution for 24 h, following the acclimatization the bottles were removed for 12 h, and then one bottle of 1% sucrose solution bottles reintroduced alongside the water bottle for 24 h. The sucrose preference was calculated as:

$$ {\mathrm{Sucrose}}\:{\mathrm{preference}}\% = \left( {\frac{{{\mathrm{Total}}\:{\mathrm{Sucrose}}\:{\mathrm{Intake}}\:\left( V \right)}}{{{\mathrm{Total}}\:{\mathrm{sucrose}}\:{\mathrm{intake}}\:\left( V \right) + \:{\mathrm{Water}}\left( V \right)}}} \right)*100 $$

#### Anxiety index, and emotionality Z-score calculation

The Z-normalization methodology was used to gain comprehensive and integrated behavioral measures form the EPM and OF test. The directionality of the Z-scores was adjusted so that decreased-negative Z-score values reflected a high level of emotionality. Furthermore, an anxiety index in the EPM was also calculated: the index combines EPM parameters into one unified ratio with values ranging from 0 to 1, with a higher value indicating an increased anxiety level [79].

### Gene expression analysis

The RNA was extracted from brain samples by combining TRI reagent (Sigma-Aldrich, St. Louis, MO, USA) and Purelink-TM kit (Thermo Fisher Scientific, Waltham, MA, USA). cDNA was prepared, and real-time PCR was performed as described before [80]. The relative expression of the target gene normalized to β-actin and calculated using the ΔΔCt method (Primers are depicted in Table 2).

**Table 2:** The gene primer sequences

Target Gene	Sequence
C-fos	Forward: ACGGAGAATCCGAAGGGAAAGGAA Reverse: TCTGCAACGCAGAC TTCTCGTCTT
GAD 65	Forward: GGCTCTGGCTTTTGGTCCTTC Reverse: TGCCAATTCCCAATTATACTCTTGA
GAD 67	Forward: GCTGGAAGGCATGGAAGGTTTTAA Reverse: AATATCCCATCACCATCTTTATTTGACC
β-actin	Forward: GACGTTGACATCCGTAAAGACC Reverse: CTAGGAGCCAGGGCAGTAATCT

### Biochemistry measurements

Corticosterone level was determined in serum samples. Briefly, blood samples were collected following two weeks of CUS, at the same time-point (16:00 pm), and centrifuged for 15 min at 1,500*g* at 4 °C. Serum samples were stored at − 80 °C. Corticosterone levels were determined using the Enzyme-linked immunoassay kit (KGE009, R&D Systems) according to the manufacturer’s instructions.

### Data analysis

Statistical analyses were performed using IBM SPSS statistics (v26), and GraphPad Prism (v10). All data were expressed as Mean ± SEM. Differences between groups were assessed by independent sample t*-*test, one-way ANOVA. Changes in mechanical sensitivity were assessed using mixed-model repeated-measures analysis of variance (GLM), and Paired sample t-test. The effects of stress (Non-CUS or CUS) and vulvar injection (Saline or Zymosan) were assessed by two-way ANOVA. Significant main effects and interactions were further pursued using Post hoc by Tukey’s test. Pearson’s correlation coefficient was used to examine correlation between variables. The accepted significance value for all tests was set at *P* < 0.05.

## Data Availability

The datasets included in the study and the code for statistical analysis are available from the corresponding author upon request.
